# Trends in energy and macronutrient intake among Taiwanese older adults in 1999–2000, 2005–2008 and 2013–2016 periods

**DOI:** 10.1186/s12889-023-15810-9

**Published:** 2023-05-12

**Authors:** Chih-Hsueh Lin, Hsing-Yi Chang, Tsai-Chung Li, Chiu-Shong Liu, Wen-Yuan Lin, Meng-Chih Lee, Li-Na Liao, Chia-Ing Li, Chih-Yi Hsiao, Hsin-Ling Fang, Wen-Tzu Wu, Cheng-Chieh Lin

**Affiliations:** 1 School of Medicine, College of Medicine, China Medical University, No. 100, Sec. 1, Jingmao Rd, Beitun Dist., Taichung City, 406040 Taiwan, Republic of China; 2 Department of Family Medicine, China Medical University Hospital, Taichung, Taiwan; 3 Department of Geriatric Medicine, China Medical University Hospital, Taichung, Taiwan; 4 Institute of Population Health Sciences, National Health Research Institutes, Miaoli, Taiwan; 5 Department of Public Health, College of Public Health, China Medical University, Taichung, Taiwan; 6 Department of Family Medicine, Taichung Hospital, Ministry of Health and Welfare, Taichung, Taiwan; 7 Department of Medicine, National Cheng Kung University, Tainan, Taiwan; 8 Department of Medical Research, China Medical University Hospital, Taichung, Taiwan; 9 Department of Food Nutrition and Health Biotechnology, Asia University, Taichung, Taiwan

**Keywords:** Older adults, Nutrient, Balanced diet

## Abstract

**Background:**

This study aimed to explore trends, in 3 periods, in the intake of energy and macronutrients among Taiwanese older adults.

**Methods:**

Study subjects were those aged ≥65 years in the Nutrition and Health Survey in Taiwan 1999–2000 as well as the surveys in 2005–2008 and 2013–2016. Twenty-four-hour dietary recall data were obtained. This study used the 3 nutrition survey datasets for 1999–2000, 2005–2008, and 2013–2016, including data on the questionnaire, physical examination, and dietary intakes. Each nutrition survey involved the face-to-face household interview, and individual’s dietary intake of carbohydrate, fat, and protein (% of energy) was estimated. Subsequently, intake statuses of the three macronutrients were classified into below, meeting, and above intake categories.

**Results:**

In the 2013–2016 survey, approximately 40% of the older adults had a low intake of energy. The prevalence of older adults with a meeting intake of carbohydrate, fat, and protein have increased from the 1999–2000 to 2013–2016 periods. The prevalence of people having a low intake of carbohydrate declined from the 1999–2000 period to the 2013–2016 period. The prevalence of high fat intake in 2013–2016 was approximately 5% higher than that in 1999–2000. In the 2013–2016 period, the prevalence of low intake of carbohydrate, fat, and protein were 25.9, 24.5, and 4.9%, respectively; moreover, the prevalence of high intake of the aforementioned macronutrients were 38.7, 36.2, and 17.6%, respectively.

**Conclusions:**

Our study provides important evidence on the dietary patterns, as well as their changes over time among Taiwanese older adults. Such information would be useful for health policy makers about the burden of unbalanced diet and for nutrition educators on planning nutrition promotion interventions about well-balanced dietary for the older persons.

## Background

Aging is itself a process of gradual malnutrition. Malnutrition results in the onset of anemia as well as the deterioration of bone density, body functions, particularly muscle mass and cognitive function [1]. According to the Taiwan Longitudinal Study on Aging, among older adults in Taiwan, malnutrition has a prevalence of 1.7% for men and 2.4% for women [2]. In a review article of 21 studies, Guigoz et al. reported that the prevalence of malnutrition and low nutrition among older adults was approximately 2% and almost 24%, respectively [3]. Furthermore, for older adults who appear healthy, their nutrition intakes are far less likely to comply with dietary recommendations. Studies on the Dutch population have indicated that for older adults aged 74–79 who ingested over 1500 kcal of energy per day, 19% of men and 26% of women had a low intake of at least one micronutrient [4]. A survey of community-dwelling older people in United states reported that almost 40% of older adults consumed less than their recommended daily intake of protein [5]. Among older adults recruited from the New York City community centers, 67% of women and 50% of men did not meet the recommended dietary allowance for protein [6]. The malnutrition and low nutrition in older adult population are important health issue.

Risk factors of malnutrition in older adults are multifactorial and include social, physiological and economic parameters commonly referred to as dementia, dysgeusia, dysphagia, diarrhea, depression, disease, poor dentition, functional impairment, and medications [7]. According the findings from longitudinal studies of community-dwelling older adults, the predictors of malnutrition, included poor appetite, self-reported poor health, aging, poor physical function, polypharmacy, dependence on activities of daily living, difficulty walking or climbing stairs, oral health problems, and hospitalization [8–11].

Decreasing weight is a symptom of malnutrition, and the death risk of underweight older adults was 2.05 times the risk of older adults with a healthy weight [12]. Among older adults, the insufficient intake of nutrients directly and indirectly increases the risk of mortality [13]; these indirect increases include diminished cognition from a lack of vitamin B [14]. Older adults with an insufficient intake of protein are prone to sarcopenia [15]. Cognition and muscle mass are significantly correlated with mortality risk [16, 17]. In addition, studies have shown nutritional status to be an important predictor of depression among older adults. If older adults do not perceive eating to be enjoyable, they are more prone to depression, resulting in symptoms such as poorer nutrition and weight loss [18]. Nutritional conditions, particularly a deficiency or excess of nutrients, can induce or directly result in acute cognitive dysfunction [19, 20]. A longitudinal study on the population of Taiwan has also indicated that older adults with poor nutrition (Mini Nutritional Assessment score ≤ 23.5 points) have a 43% higher risk of cognitive decline after 4 years [21]. Among older adults, malnutrition causes various diseases and dysfunctions, which further imposes a large medical healthcare burden. Consequently, nutrition among older adults is key to maintaining their health and the first step to preventing disabilities.

Studies on the prevalence of low and high intakes for the three macronutrients, including carbohydrate, fat, and protein among older adults in Taiwan remain inadequate. Most studies have been individual reports limited to a survey, and studies connecting various surveys have been rarely conducted. Low or high intakes represent abnormal dietary patterns and less balanced diets. Prevalence studies of abnormal dietary patterns can inform researcher, and guide policy-makers about its burden to identify the priorities in nutritional issues for older persons. In addition, these prevalence studies can provide data for informing the assessment of interventions. Therefore, to understand the trend of relative prevalence of nutrition intake of older adults in the last 15 years, we used the 3 waves of nutrition surveys of the 1999–2000 Elderly Nutrition and Health Survey in Taiwan (Elderly NAHSIT 1999–2000), 2005–5008 Nutrition and Health Survey in Taiwan (NAHSIT 2005–2008), and the NAHSIT 2013–2016. Specifically, this study analyzed the intake conditions of the three macronutrients among older adults in Taiwan during the 3 periods of 1999–2000, 2005–2008, and 2013–2016.

## Methods

### Study population

The major sources of information in this study were the Elderly NAHSIT 1999–2000 as well as the NAHSIT 2005–2008 and NAHSIT 2013–2016 surveys. Parts of the database were from the Health and Welfare Data Science Center, Taiwan. The target population of the Elderly NAHSIT 1999–2000 was Taiwanese citizens at least 65 years old on January 1, 1999, excluding institutionalized residents including hospitals, nursing homes, other institutions, etc. at the time when the list of eligible participants was drafted [22]. A stratified multistage cluster sampling was adopted as the sampling method. In total, 1937 people were enrolled in the Elderly NAHSIT 1999–2000. Finally, a total of 1840 people were included in this study analysis after excluding those whose data were missing or with extreme intake values.

The target population of the NAHSIT 2005–2008 was Taiwanese citizens who were either 0–6 years old or at least 19 years old [23], excluding pregnant women, lactating women, and institutionalized residents, as well as patients who had dementia, notifiable diseases, or severe illnesses. The survey’s sampling method was a stratified 3-stage sampling approach. Five mutually-exclusive strata based on geographical location and population density, as well as three extra strata for specific population groups, were adopted. Then, 3-stage probability sampling was used to select the sample within each stratum. In the first sage, 48 townships and city districts were sampled. In the second stage, 2 sampling blocks were sampled from each of the selected townships and city districts. In the third stage, 64 people were sampled from each sampling block. Finally, 6189 people participated in this survey. This study only analyzed the NAHSIT data on those at least 65 years old (1547 people).

The target population of the NAHSIT 2013–2016 was Taiwanese citizens older than 2 months living in either Penghu county or the island of Taiwan—with 20 counties and cities [24]. A stratified multistage clustering sampling design was used. First, each county/city was considered a stratum. Then, each stratum was further classified into 2 substrata according to the degree urbanization. In each substratum, the villages/Lis within townships/city districts were sorted from high to low according to their population densities. Subsequently, using the probability proportional to size sampling methods, a total of 40 villages/Li were selected for each survey year for the first stage. At the second stage of sampling, 10 households were selected for starting point, then systematic sampling approach was used to select households and household members who were eligible were interviewed. Finally, 11,072 people participated in this survey. This study only analyzed the data of older adults at least 65 years old (1920 people). The three national surveys were all led by the Taiwan government and conducted by the same team, so the collection methods for the three surveys are similar. Although the items of the food frequency questionnaire varied according to the eating habits at the time of the survey, the validation of the questionnaires has been published previously [25]. The Ethical Review Board of China Medical University Hospital approved the study protocol (CMUH105-REC2–032).

### Study variables

This study used the 3 nutrition survey datasets for 1999–2000, 2005–2008, and 2013–2016, including data on the questionnaire, physical examination, and dietary intakes. Each nutrition survey involved the face-to-face household interview and the mobile health examination. Our analytical dimensions included demographic characteristics, in addition to the dietary nutrient intake of participants.

Demographic characteristics included those on sex, age, education level, marriage status, personal monthly income, and residential urbanization. Data on health behavior included those on exercise, smoking status, and alcohol drinking status. Medical history data included those for type 2 diabetes, hypertension, heart disease, hyperlipidemia, stroke, cancer, chronic kidney disease, and fracture. Cognitive function was assessed by using the Short Portable Mental Status Questionnaire (SPMSQ) in the two datasets (1999–2000 and 2005–2008), and the Mini-Mental State Examination (MMSE) in the other dataset (2013–2016). The SPMSQ is a 10-item questionnaire with total scores ranging from 0 to 10 and a cut-off score for subthreshold abnormal cognitive function of  ≥ 3 [26]. The abnormal cognitive function in 2013–2016 was not provided because the data of MMSE was not obtained in this study. Anthropometric measurements included those on height, weight, body waist circumference, and blood pressure. Body mass index (BMI) was calculated as weight divided by height squared (kg/m^2^). All subjects were categorized into four groups: < 18.5 (underweight), 18.5–23.9 (normal), 24–26.9 (overweight) and ≥ 27 kg/m^2^ (obese) by the definition proposed by the Health Promotion Administration in Taiwan [27].

#### Dietary assessment

Dietary intake data were collected by using the questionnaire of 24-hour dietary recall (including household recipes, individual dietary recall, etc.), then nutrient intakes were computed. Methods of the 24-hour dietary recall and nutrient computation were identical to that employed in NAHSIT 1993–1996 [28]. In NAHSIT 2005–2008, the Nutrients and food sources were divided into 12 major and 47 minor items in order to assess nutrient intakes from each food group. The NAHSIT 2013–2016 used a 79-item food-frequency questionnaire (FFQ) to appraise the dietary status of the preceding year, and the food items were merged into 23 food groups based on their characteristics and nutrient contents. The FFQ of the Elderly NAHSIT 1999–2000 is a simplified version covering 16 items. Validation of a similar simplified FFQ with previous measurement has been previously published [25]. For all items the frequency of intake per week was measured.

Data on calorie intake and dietary pattern were obtained from 24-hour dietary recall by trained interviewers. Dietary behavior was assessed by an index: dietary diversity scores (DDS, range 0–6), a count of food groups [29, 30]. A DDS score of 1 was defined as ingestion at least half a serving per day for a food group. The DDS comprised six major food groups, including cereals/grains, vegetables, fruits, fats/oils, dairy products, and soybean/fish/eggs/meat which from the 24-hour dietary recall.

To analyze the dietary intake patterns of participants, we considered the three macronutrients, including dietary carbohydrate, fat, and protein, which were derived from 24-hour dietary recall and presented as the percentage of total energy intake. The food data of a single 24-hour dietary recall were linked with nutrient information from a food composition database to analyze the macronutrient content. The macronutrient and energy intakes from each food item were then derived. The total daily energy intake was derived by multiplying the amount of consumption of each item by its caloric content per serving and then totaling the caloric intake for all food items. The percentage of total kilocalories from carbohydrate, protein, and fat intakes was then calculated. The recommended daily allowance for carbohydrates is 50–60% of dietary energy from carbohydrates [31]. If an individual consumed at this range, the dietary energy from carbohydrate status was classified as meeting, if lower than 50%, as below, if higher than 60%, as above. The recommended daily ranges of intakes for fats and proteins were 20–30% and 10–20% of energy, respectively [31] and the status for the dietary energy from fats and proteins was defined in the similar way. Nutrient intakes data included energy and protein. Nutrient intakes were compared to the dietary reference intakes (DRIs, version 7) [32]. Intakes from the six major food groups, including cereals/grains, vegetables, fruits, fats/oils, dairy products, and soybean/fish/eggs/meat were compared to the Dietary Guidelines for the Taiwanese older persons [31]. To estimate the prevalence for insufficient intake of a given nutrient or food group, first, we calculated the ratio of an individual’s intake to the recommended daily allowance based on the individual’s age and sex category. Subsequently, this ratio of less than 0.75 represented insufficient intake for the nutrient or food group [33, 34].

### Statistical analysis

In the statistical analysis, the data were segmented by the 3 survey periods. Continuous variables were characterized by their mean ± standard error (SE), and categorical variables were characterized by variations in the number of people (in percentage). All analyses were weighted and adjusted to obtain population-representative estimates and SEs. The linear trend analysis was used in analyzing the trends of characteristics distribution of study subjects across the three surveys. The non-binary categorical variables (ex. age groups, education levels) were treated as binary categorical variables before using the trend test. Since low BMI is often considered a criterion for diagnosing malnutrition, trends in underweight were further analyzed. To compare the prevalence of underweight in the 3 surveys, the standardized morbidity ratio (SMR) was adopted and the NAHSIT 1999–2000 served as the reference period. Poisson regression was used to examine the trend of the SMR for underweight after adjusting for age, personal monthly income, and urbanization. As Elderly NAHSIT 1999–2000, NAHSIT 2005–2008, and NAHSIT 2013–2016 all used a stratified, multistage probability design, statistical analyses were conducted using the statistical package SAS for Windows (Version 9.4, SAS, Cary, NC, USA) and the SUrvey DAta ANalysis (SUDAAN) to account for sampling schemes. The SUDAAN software was used to weight the three survey samples and examine the overall trend and trend stratified by gender for the 3 survey periods.

## Results

### Demographic characteristics

The proportions of men in the 3 survey periods (1999–2000, 2005–2008, and 2013–2016) were 53.3, 49.7, and 46.7%, respectively (Table 1). In the aforementioned 3 periods, 33.8, 42.3, and 44.4% of respondents were older than 75 years, respectively; 23.4–39.2% had junior high school education and higher; and 24.5, 19.0, and 33.0% had a monthly income more than NT$10,000, respectively. Furthermore, relative to the 1999–2000 period, the other two NAHSIT surveys had more respondents living in rural areas (66.8 and 62.3%). As for health behavior, 54–62% of older adults in the 3 surveys had the habit of exercising. For the 3 aforementioned surveys, the proportions of respondents with a smoking habit declined over the survey periods (trend test, *p* < 0.05), at 21.7, 12.5, and 8.9%, respectively, and that for those with a drinking habit slightly increased over the survey periods (trend test, p < 0.05), at 18.5, 21.5, and 25.3%, respectively. Approximately 35–43% of older adults had poor dietary diversity (DDS ≤ 4). The prevalence of abnormal cognitive function in two surveys were approximately 11–13% (Lack of result in NAHSIT 2013–2016). The averages BMI of the 3 surveys were 23–25 kg/m^2^. For the NAHSIT 2013–2016, the prevalence of type 2 diabetes, hypertension, heart disease, hyperlipidemia, and fracture were 23.3, 53.6, 14.4, 13.4, and 25.4%, respectively.

**Table 1:** Characteristics distribution of study subjects

Variables	1999–2000		2005–2008		2013–2016		Trend test *P*-value^b^
	n/mean	%/SE^a^	n/mean	%/SE^a^	n/mean	%/SE^a^	
Sample size of questionnaire	1840		1547		1920		
Demographic characteristics							
Sex							0.845
Men	925	53.3	770	49.7	959	46.7	
Women	915	46.7	777	50.3	961	53.3	
Age (years)	72.8	0.25	73.5	0.24	74.2	0.27	< 0.001
65–69	718	35.8	486	32.1	638	30.6	< 0.001
70–74	605	30.5	445	25.7	513	24.9	
75–79	329	19.0	366	25.9	382	19.3	
≥ 80	188	14.8	250	16.4	387	25.1	
Education							< 0.001
Illiterate	640	36.7	413	26.5	289	14.6	
≤ 6 years	814	40.0	776	46.2	902	46.2	
> 6 years	379	23.4	356	27.3	725	39.2	
Marital status							0.798
Single/divorced/widowed/separated	621	33.8	558	34.7	641	35.4	
Married/cohabitation	1210	66.2	984	65.3	1278	64.6	
Personal monthly income							< 0.001
< NTD 10,000	1329	75.5	1237	81.0	1210	67.0	
≥ NTD 10,000	379	24.5	249	19.0	614	33.0	
Urbanization							< 0.001
Urban areas	843	55.9	348	33.2	536	37.7	
Rural areas	997	44.1	1199	66.8	1384	62.3	
Health behaviors							
Exercise							0.014
No	895	44.9	587	37.9	868	46.0	
Yes	932	55.1	960	62.2	1051	54.0	
Smoking status							0.014
No	1197	63.0	930	69.8	1321	71.1	
Ever	233	15.3	260	17.7	371	20.0	
Yes	397	21.7	190	12.5	178	8.9	
Alcohol drinking							< 0.001
No	1347	74.8	902	68.0	1063	67.6	
Ever	124	6.7	169	10.5	126	7.1	
Yes	363	18.5	307	21.5	413	25.3	
Dietary diversity score							< 0.001
> 4	963	57.1	857	57.9	1211	65.0	
≤ 4	877	42.9	688	42.1	708	35.0	
Cognitive function							< 0.001
Normal	1558	87.5	1162	89.0	–	–	
Abnormal	240	12.5	174	11.0	–	–	
History of diseases							
Type 2 diabetes	316	16.6	348	22.3	451	23.3	< 0.001
Hypertension	857	46.3	820	51.7	1036	53.6	< 0.001
Heart disease	350	19.3	269	17.3	287	14.4	< 0.001
Hyperlipidemia	137	8.1	188	13.9	234	13.4	< 0.001
Stroke	92	5.6	89	4.9	93	4.4	0.818
Cancer	40	2.3	47	3.5	91	4.6	< 0.001
Chronic kidney disease	67	3.4	44	2.7	70	3.3	0.981
Fracture	266	15.6	274	19.9	469	25.4	< 0.001
Sample size of physical examination	1840		1023		1440		
Anthropometric measurements							
BMI (kg/m^2^)	23.7	0.17	24.6	0.15	24.7	0.13	0.029
< 18.5	97	7.1	34	2.5	28	2.1	< 0.001
18.5–23.9	625	47.9	373	41.6	444	42.4	
24–26.9	413	28.0	314	33.6	303	32.1	
≥ 27	251	17.0	226	22.3	258	23.4	
Systolic blood pressure (mmHg)	134.4	0.98	129.4	0.88	129.3	0.85	0.001
Diastolic blood pressure (mmHg)	75.1	0.57	71.7	0.58	73.2	0.46	< 0.001
Waist circumference (cm)	83.8	0.31	86.5	0.41	88.2	0.32	< 0.001

-No information provided because data was not available at the time of analysis in this study

^a^ Weighted adjusted

^b^ Trend test for the proportions of participants who were aged ≥75 years, illiterate, no smoking, no alcohol drinking, and BMI ≥ 27 kg/m^2^

### Dietary intake patterns of the three macronutrients

Each individual’s dietary intake of carbohydrate, fat, and protein (% of energy) was estimated. According to the newest 2013–2016 NAHSIT survey, the prevalence of relative macronutrient intakes below the recommended daily allowance were 25.9% for carbohydrates, 24.5% for fats, and 4.9% for proteins (Table 2). A further stratified analysis revealed that for both men and women in 3 waves of survey, the prevalence of relative macronutrient intakes meeting the recommended daily allowance were increasing over the survey periods. The prevalence of meeting intake for carbohydrate increased from 28.4 to 35.6% for men and increased from 23.5 to 35.1% for women. The prevalence of normal intake for fat increased from 33.1 to 37.1% for men and increased from 28.8 to 41.2% for women. The prevalence of meeting intake for protein increased from 66.7 to 78.0% for men and increased from 67.3 to 76.9% for women. We also observed that the prevalence of above fat intake for men significantly increased over the 3 periods, and the prevalence of above protein intake for both men and women significant decreased over the 3 periods.

**Table 2:** Intake patterns of macronutrients

Intake status^a^	Overall				Men				Women			
	1999–2000	2005–2008	2013–2016	Trend test *P*-value	1999–2000	2005–2008	2013–2016	Trend test *P*-value	1999–2000	2005–2008	2013–2016	Trend test *P*-value
	(*n* = 1840)	(*n* = 1545)	(*n* = 1919)		(*n* = 925)	(*n* = 769)	(*n* = 959)		(*n* = 915)	(*n* = 776)	(*n* = 960)	
**Carbohydrate**				< 0.001				0.070				< 0.001
Below (< 50%)	28.2	30.8	25.9		30.1	33.9	30.3		26.0	27.8	22.1	
Meeting (50–60%)	26.1	31.9	35.4		28.4	32.2	35.6		23.5	31.6	35.1	
Above (> 60%)	45.7	37.3	38.7		41.5	34.0	34.1		50.5	40.6	42.8	
**Fat**				< 0.001				0.004				< 0.001
Below (< 20%)	37.6	26.8	24.5		33.4	25.2	21.7		42.4	28.3	27.0	
Meeting (20–30%)	31.1	33.8	39.3		33.1	33.3	37.1		28.8	34.3	41.2	
Above (> 30%)	31.3	39.4	36.2		33.5	41.5	41.2		28.8	37.4	31.8	
**Protein**				0.002				0.004				0.009
Below (< 10%)	8.3	6.6	4.9		7.5	5.1	5.2		9.1	8.1	4.7	
Meeting (10–20%)	67.0	71.0	77.4		66.7	71.2	78.0		67.3	70.8	76.9	
Above (> 20%)	24.8	22.4	17.6		25.8	23.7	16.8		23.7	21.1	18.4	

^a^ Weighted adjusted

In a subsequent analysis, the people were divided into two groups based on their total energy intake was sufficient for comparison of the distributions for their three macronutrients intake. The distributions of the fat and carbohydrate intakes of the two groups significantly differed, and both groups had various problems with regard to unbalanced diets. In all 3 surveys, for those with below intake of total energy, the prevalence of above carbohydrate was higher than that for those with sufficient total energy intake but the prevalence of above fat was much lower for those with below intake of total energy than that for those with sufficient total energy intake (Table 3). For a further stratified analysis by sex, men and women had the same trends as the overall population, as detailed in Table 4.

**Table 3:** Comparisons of distributions for intake patterns of macronutrients between those who had sufficient and insufficient total energy intake stratified by three periods

Intake status^a^	Overall								
	1999–2000			2005–2008			2013–2016		
	Insufficient energy intake	Sufficient energy intake	*P*-value	Insufficient energy intake	Sufficient energy intake	*P*-value	Insufficient energy intake	Sufficient energy intake	*P*-value
	(*n* = 928)	(*n* = 912)		(*n* = 887)	(*n* = 658)		(*n* = 789)	(*n* = 1130)	
**Carbohydrate**			< 0.001			0.001			< 0.001
Below (< 50%)	19.4	37.7		24.8	38.1		18.3	30.9	
Meeting (50–60%)	24.8	27.6		31.7	32.2		31.3	38.0	
Above (> 60%)	55.8	34.8		43.5	29.7		50.5	31.1	
**Fat**			< 0.001			< 0.001			< 0.001
Below (< 20%)	46.9	27.6		32.5	19.6		35.1	17.7	
Meeting (20–30%)	31.4	30.8		36.3	30.8		39.7	39.0	
Above (> 30%)	21.8	41.7		31.2	49.6		25.3	43.3	
**Protein**			0.025			0.200			0.005
Below (< 10%)	5.4	11.3		7.9	5.1		7.4	3.4	
Meeting (10–20%)	68.7	65.1		69.5	72.8		74.1	79.6	
Above (> 20%)	25.9	23.6		22.6	22.1		18.6	17.0	

^a^ Weighted adjusted

**Table 4:** Stratified analysis by gender and survey period: comparing intake patterns of macronutrients between those who had sufficient and insufficient total energy intake

	Men									Women								
	1999–2000			2005–2008			2013–2016			1999–2000			2005–2008			2013–2016		
Intake status ^a^	Insufficient energy intake	Sufficient energy intake	*P*-value	Insufficient energy intake	Sufficient energy intake	*P*-value	Insufficient energy intake	Sufficient energy intake	*P*-value	Insufficient energy intake	Sufficient energy intake	*P*-value	Insufficient energy intake	Sufficient energy intake	*P*-value	Insufficient energy intake	Sufficient energy intake	*P*-value
	(*n* = 461)	(*n* = 464)		(*n* = 427)	(*n* = 342)		(*n* = 379)	(*n* = 580)		(*n* = 467)	(*n* = 448)		(*n* = 460)	(*n* = 316)		(*n* = 410)	(*n* = 550)	
Carbohydrate			< 0.001			0.034			0.011			< 0.001			0.003			< 0.001
Below (< 50%)	23.3	37.5		28.8	39.3		22.6	34.8		15.0	37.9		21.3	36.8		15.0	27.1	
Normal (50–60%)	28.8	28.1		30.2	34.3		34.7	36.2		20.2	27.0		32.9	29.8		28.6	39.8	
Above (> 60%)	48.0	34.5		40.9	26.4		42.8	29.1		64.8	35.1		45.8	33.4		56.3	33.0	
Fat			0.001			< 0.001			0.001			< 0.001			0.001			< 0.001
Below (< 20%)	38.5	27.8		31.2	18.8		29.0	17.5		56.5	27.3		33.8	20.6		39.7	17.8	
Normal (20–30%)	36.2	29.8		37.0	29.3		41.8	34.4		25.9	31.9		35.6	32.6		38.0	43.6	
Above (> 30%)	25.3	42.4		31.9	52.0		29.2	48.2		17.7	40.8		30.6	46.9		22.3	38.6	
Protein			0.030			0.080			0.468			0.103			0.232			0.020
Below (< 10%)	4.6	10.7		6.4	3.7		6.8	4.2		6.4	12.0		9.2	6.7		7.8	2.5	
Normal (10–20%)	67.1	66.3		67.3	75.4		77.4	78.4		70.5	63.8		71.5	69.8		71.5	80.9	
Above (> 20%)	28.3	23.0		26.3	20.9		15.8	17.4		23.2	24.2		19.3	23.6		20.7	16.6	

^a^ Weighted adjusted

### Prevalence of the insufficient intake of nutrients and of six major food groups

In 1999–2000, 52.1% of older adults had an insufficient intake of energy, and this prevalence decreased over survey periods and became 39.4% in 2013–2016 (Table 5). Over the three survey periods, the top 3 insufficient intakes of food groups were all the same: dairy products, fruits, and fats/oils (Table 5). We observed there was an increasing prevalence in insufficient intake of dairy products over the survey periods (*P* < 0.01 for overall and stratified by sex). On the contrary, there was no significant trend for prevalence in insufficient intake of fruits, and fats/oils. In the newest survey (2013–2016 NAHSIT), the prevalence of insufficient intake of dairy products (81.8%) and fruits (72.2%) of the six major food groups reached approximately 70–80%. A further stratified analysis by sex revealed that for both men and women, the prevalence of insufficient intake of dairy products reached 80%. For vegetables and fruits, the prevalence of insufficient intake were higher for men than women. For cereals/grains, protein, and fats/oils, the prevalence of insufficient intake were higher for women than men.

**Table 5:** Prevalence of insufficient intake for energy, protein and six major food groups

Prevalence of insufficient intake^a^	Overall				Men				Women			
	1999–2000 (*n* = 1840)	2005–2008 (*n* = 1545)	2013–2016 (*n* = 1919)	Trend test *P*-value	1999–2000 (*n* = 925)	2005–2008 (*n* = 769)	2013–2016 (*n* = 959)	Trend test *P*-value	1999–2000 (*n* = 915)	2005–2008 (*n* = 776)	2013–2016 (*n* = 960)	Trend test *P*-value
Energy	52.19	55.2	39.4	< 0.001	52.3	52.1	36.5	< 0.001	51.9	58.2	42.0	0.003
Protein	24.8	26.1	18.2	0.001	21.8	20.3	13.3	0.006	28.2	31.7	22.4	0.017
**Six major food groups**												
Cereals/grains	47.0	59.0	37.7	< 0.001	45.5	57.8	33.7	< 0.001	48.7	60.2	41.2	< 0.001
Soybean/fish/eggs/meat	58.1	53.2	45.7	< 0.001	53.6	47.4	41.9	0.009	63.3	59.1	49.0	0.001
Dairy products	71.9	75.8	81.8	0.001	72.2	74.5	82.4	0.002	71.5	77.0	81.2	0.006
Vegetables	55.5	50.0	54.5	0.110	60.4	56.7	59.0	0.614	50.0	43.5	50.6	0.069
Fruits	77.4	75.0	72.2	0.082	84.3	79.2	82.4	0.274	69.6	70.9	63.2	0.041
Fats/oils	73.8	69.2	66.8	0.115	73.6	67.8	63.8	0.034	74.1	70.6	69.4	0.419

Insufficient intake: nutrient adequacy ratio < 75%

^a^ Weighted adjusted

### Standardized morbidity ratios of underweight

Using the 1999–2000 survey as a reference for comparison, we calculated the SMRs of underweight of the two other surveys. After adjusting for sex, age, personal monthly income, and urbanization, the SMRs of underweight were declining over the survey periods (*p* < 0.001) (Table 6). The prevalence of underweight in older adults was 7.1% in 1999–2000, and it decreased to 2.1% in 2013–2016; the SMR was 0.37 (95% CI: 0.24–0.56). In a further stratified analysis, the ratio also declined for men and women (*p* < 0.001 and *p* = 0.003). The prevalence of underweight among male older adults decreased from 7.2% in 1999–2000 to 1.4% in 2013–2016, with the SMR of 0.31 (95% CI: 0.16–0.57). The prevalence of underweight among female older adults decreased from 7.0 to 2.8%, with the SMR of 0.44 (95% CI: 0.24–0.80).

**Table 6:** Standardized morbidity ratios of underweight

Underweight	1999–2000		2005–2008			2013–2016			Trend test *P*-value^c^
	Prevalence n (%)^a^	Total	Prevalence n (%)^a^	Total	SMR (95% CI)^b^	Prevalence n (%)^a^	Total	SMR (95% CI)^b^	
Overall	97 (7.1)	1386	34 (2.6)	938	0.49 (0.33, 0.74)	28 (2.1)	1033	0.37 (0.24, 0.56)	< 0.001
Men	49 (7.2)	713	19 (3.1)	493	0.49 (0.28, 0.84)	13 (1.4)	540	0.31 (0.16, 0.57)	< 0.001
Women	48 (7.0)	712	15 (2.1)	445	0.49 (0.27, 0.89)	15 (2.8)	493	0.44 (0.24, 0.80)	0.003

*Underweight *BMI < 18.5 kg/m^2^, *SMR *standardized morbidity ratio

^a^ Weighted adjusted

^b^ Using Poisson regression with adjustment of sex, age, personal monthly income, and urbanization; under gender stratified analysis, adjustment with age, personal monthly income, and urbanization

^c^ Trend test of survey period

## Discussion

According to the NAHSIT 1999–2000 and 2005–2008 surveys, for both men and women, the mean intakes of total energy were slightly declining across the survey periods (for men: from 1829 kcal to 1711 kcal; for women: from 172 kcal to 1316 kcal) [35]. Our analysis of the 3 surveys indicated that the proportions of people not reaching 75% of recommended total energy intake increased from 52.1% in 1999–2000 to 55.2% in 2005–2008 but decreased to 39.4% in 2013–2016. This indicates that although the total energy intake of older adults has increased in recent years, approximately 40% of older adults still have an insufficient intake of energy. Based on the energy from carbohydrates, fats, and proteins, regardless of sufficient total energy intake or not, the proportions of normal intake of the three macronutrients have been increasing over time. The proportions of people having a normal intake of carbohydrate, fat, and protein increased from 26.1 to 35.4%, 31.1 to 39.3%, and 67.0 to 77.4%, respectively, indicating that dietary behavior is increasingly valued and being practiced.

The proportions of those having a meeting intake of the three macronutrients have been increasing through the survey years, but the proportion of those having an insufficient intake of energy still reached approximately 40%. Therefore, the older adults should be informed the intake quantity of the three macronutrients needed to increase. In addition, malnutrition is a cause of many diseases. The proportion of calories from carbohydrates increased between 2005 and 2008 and 2013–2016 surveys in this study. The similar pattern was found during earlier time periods in American (from 1977 to 2010) [36], but the daily consumption of carbohydrates decreased from 2011 to 2018 [37]. However, in this study, about 25% of older adults in the 2013–2016 survey did not eat enough carbohydrates, and 40% ate too much carbohydrates. The excessive glucose intake is a risk for developing diabetes, and the insufficient carbohydrate intake influences the metabolism of proteins and lipids in the body, which will worsen sarcopenia in the elderly. Studies on US populations have noted that the higher daily intake of whole grains can significantly decrease risk of total mortality among middle-aged and older adults aged 50–71 years [38].

The major sources of fats are the fats/oils group. A study on US populations has noted that an increased risk of dementia was associated with high intake of total fat (relative risk = 2.4) [39], and a pooled analysis of 10 prospective cohort studies reported that high intake of total fat was associated with an increased risk of lung cancer (HR = 1.07) [40]. Buijsse et al. studied the correlations of fat intake and type 2 diabetes, discovering that the incidence rate of type 2 diabetes was correlated with how often margarine was consumed [41]. The below intake of protein in older adults were prone to sarcopenia [15]. Sarcopenia is a condition with sever muscle wasting which is common in older adults and is associated with an increased mortality risk [17]. In addition, cognitive difficulties in older adults may be partly due to inadequate protein intake [42]. This study analyzed the NAHSIT 2013–2016, and the prevalence of the insufficient intake of protein (not reaching 75% of the recommended intake) for older adults in Taiwan was 18.2%. As a percent of total energy intake, the insufficient intake of protein (< 10%) had a prevalence of 4.9%. According to a systematic review and meta-analysis (included 111 studies), the prevalence of protein-energy malnutrition for Asia, Europe, North America, South America, Australia, and African populations were approximately 5.7, 2.8, 11.5, 2.1, 5.3, and 28.4%, respectively [43]. Our analysis had definition of insufficient intake that differed from those of Crichton et al. This difference was that in our analysis, protein intake (% of total energy) was calculated from the questionnaire of 24-hour dietary recall. By contrast, Crichton et al. (2019) defined malnutrition as an MNA < 17. Our study provided the prevalence of sufficient intake for macronutrients based on 24-hour dietary recall. The major sources of protein are the soybean/fish/eggs/meat group. Several meta-analysis studies have reported the health benefits of consuming fish [44, 45] and white meat [46, 47]. The protective associations of fish consumption with all-cause mortality, fatal cardiovascular event and depression were found in two meta-analysis studies [44, 45]. An early meta-analysis study showed that the substitution of one daily serving of red meat with white meat, mainly poultry was associated with a 19% reduction of cardiovascular risk [46]. Supporting conclusions are provided by a recent meta-analysis study that reported a significant 6% reduction in all-cause mortality among subjects with the highest consumption of white meat compared to those with the lowest consumption of white meat [47].

For the six major food groups, in our analysis of the 2013–2016 data, with the exception of the cereals/grains and soybean/fish/eggs/meat groups, the prevalence of insufficient intake of the remaining four food groups were greater than 50%. Studies have indicated that the major sources of energy for older adults in Taiwan are the cereals/grains group and meat, and the intakes of the dairy products and fruits groups are significantly insufficient [48]. According to our analysis of the newest 2013–2016 data, the proportions of older Taiwanese adults have an insufficient intake of the following types of foods, from high to low: dairy products (81.8%), fruits (72.2%), fats/oils (66.8%), vegetables (54.5%), soybean/fish/eggs/meat (45.7%), and cereals/grains (37.7%). Based on the study findings from the Taiwan Longitudinal Study on Aging in 1999, the top two proportions of daily intake of food groups were the cereals/grains (98.7%) and vegetable (90.1%) groups, respectively, and the lowest one was the dairy products group (42.3%) [49]. However, this prior study didn’t consider whether the intake is sufficient or not. Our findings indicate that proportion of insufficient intake of dairy products is the most sever of the six food groups in the elderly, and was worsening over the survey years from 71.9% in the NAHSIT 1999–2000 to 81.8% in the NAHSIT 2013–2016. The main reason for recommending dairy products to the elderly is to reduce the risk of fracture by maintaining bone density through calcium intake. Subjects who avoided any dairy products had a 44% higher risk of fracture compared to those who did not avoid dairy products [50]. A diet rich in dairies was associated with a 41% lower prevalence of low bone density [51]. Furthermore, studies have also shown that an increment of 200 g of daily milk consumption can significantly decrease stroke risk by 7%, and that a daily intake of > 25 g of cheese significantly decreases stroke risk [52]. Among older adults ≥60 years old, every additional consumption of 200 g of low-fat dairy products per day can significantly decrease the risk of developing type 2 diabetes by 16% [52]. A daily intake of 80 g of yogurt can decrease the risk of developing type 2 diabetes by 14% [52]. However, the high proportion of Taiwanese people who are lactose intolerant and differences between Western and East-Asian food cultures have resulted in an increasingly severe problem of insufficient intake for dairy products.

While low BMI is commonly used to identify malnutrition [53], the prevalence of underweight (< 18.5 kg/m^2^) in the three surveys were low (< 8%) and show downward trends among older adults, including both men and women, over the last decade. In contrast, there has been a notable increase in the prevalence of overweight and obesity among older adults. However, using a 24-hour dietary recall questionnaire, it was found that about 40% of older adults had insufficient energy intake. Although overweight or obesity paradoxically poses a lower risk of mortality in the elderly population of Taiwan [54], older people with excess body weight are not spared from malnutrition. It highlights the ongoing issue of malnutrition in older population.

To the best of our knowledge, this is the first study investigating the trends of insufficient intake of nutrients and of six major food groups in three nationwide Taiwan representative samples of population aged 65 years and older for the year of 1999 to 2016. The strength of study was to increase the external validity of findings using the nationwide data, which provides representative samples of general older persons population. However, some limitations should also be noted. First, the use of cross-sectional surveys implies that the change of food intake patterns cannot describe as the change of individual behavior overtime. The interpretation of results should be careful. Second, the nonresponse of survey may affect our findings. The response rates were 55, 65 and 74% in the survey of 1999–2000, 2005–2008, and 2013–2016, respectively. The distributions of age, gender, and education were similar between respondents and nonrespondents in three surveys, except for a slight age difference in the 1999–2000 survey [22, 23]. In this study, all analyses included an adjustment for over-sampling and non-response were carried out using SUDAAN. Third, food intake data were collected in face-to-face interviews, which may have induced social desirability bias that may threatened internal validity. Last, this study did not consider confounding factors to compare the distribution of macronutrient intake patterns over the three periods. Because this is a descriptive study, we tend to report estimates of dietary status that are representative of the older population in three survey periods. In addition, this study has no authority to access the database, and it cannot be analyzed further.

## Conclusion

Our study provides important evidence on the dietary patterns and nutritional status, as well as their changes over time among Taiwanese older adults. Overall, approximately 40% of older adults had an insufficient intake of energy. Most older adults with insufficient energy intake have excessive percentages of energy from carbohydrate intake, and older adults with sufficient energy intake have the nutrition problem of having excessive percentages of energy from fat. The proportion of people who have an insufficient intake of dairy and fruits, among the 6 food groups, reached 70–80%, with this proportion for dairy significantly increasing over time.

## Data Availability

The datasets generated and/or analyzed during the current study are not publicly available due to the policy declared by Ministry of Health and Welfare in Taiwan but are available from the corresponding author on reasonable request.
